# Residues 529 to 549 participate in membrane penetration and pore-forming activity of the *Bordetella* adenylate cyclase toxin

**DOI:** 10.1038/s41598-019-42200-2

**Published:** 2019-04-08

**Authors:** Jana Roderova, Adriana Osickova, Anna Sukova, Gabriela Mikusova, Radovan Fiser, Peter Sebo, Radim Osicka, Jiri Masin

**Affiliations:** 1 Institute of Microbiology of the CAS, v.v.i., Videnska 1083, 142 20 Prague, Czech Republic; 2 Charles University, Department of Genetics and Microbiology, Faculty of Science, Vinicna 5, 128 43 Prague, Czech Republic

## Abstract

The adenylate cyclase toxin-hemolysin (CyaA, ACT or AC-Hly) of pathogenic *Bordetellae* delivers its adenylyl cyclase (AC) enzyme domain into the cytosol of host cells and catalyzes uncontrolled conversion of cellular ATP to cAMP. In parallel, the toxin forms small cation-selective pores that permeabilize target cell membrane and account for the hemolytic activity of CyaA on erythrocytes. The pore-forming domain of CyaA is predicted to consist of five transmembrane α-helices, of which the helices I, III, IV and V have previously been characterized. We examined here the α-helix II that is predicted to form between residues 529 to 549. Substitution of the glycine 531 residue by a proline selectively reduced the hemolytic capacity but did not affect the AC translocating activity of the CyaA-G531P toxin. In contrast, CyaA toxins with alanine 538 or 546 replaced by diverse residues were selectively impaired in the capacity to translocate the AC domain across cell membrane but remained fully hemolytic. Such toxins, however, formed pores in planar asolectin bilayer membranes with a very low frequency and with at least two different conducting states. The helix-breaking substitution of alanine 538 by a proline residue abolished the voltage-activated increase of membrane activity of CyaA in asolectin bilayers. These results reveal that the predicted α-helix comprising the residues 529 to 549 plays a key role in CyaA penetration into the target plasma membrane and pore-forming activity of the toxin.

## Introduction

**Figure 1:**
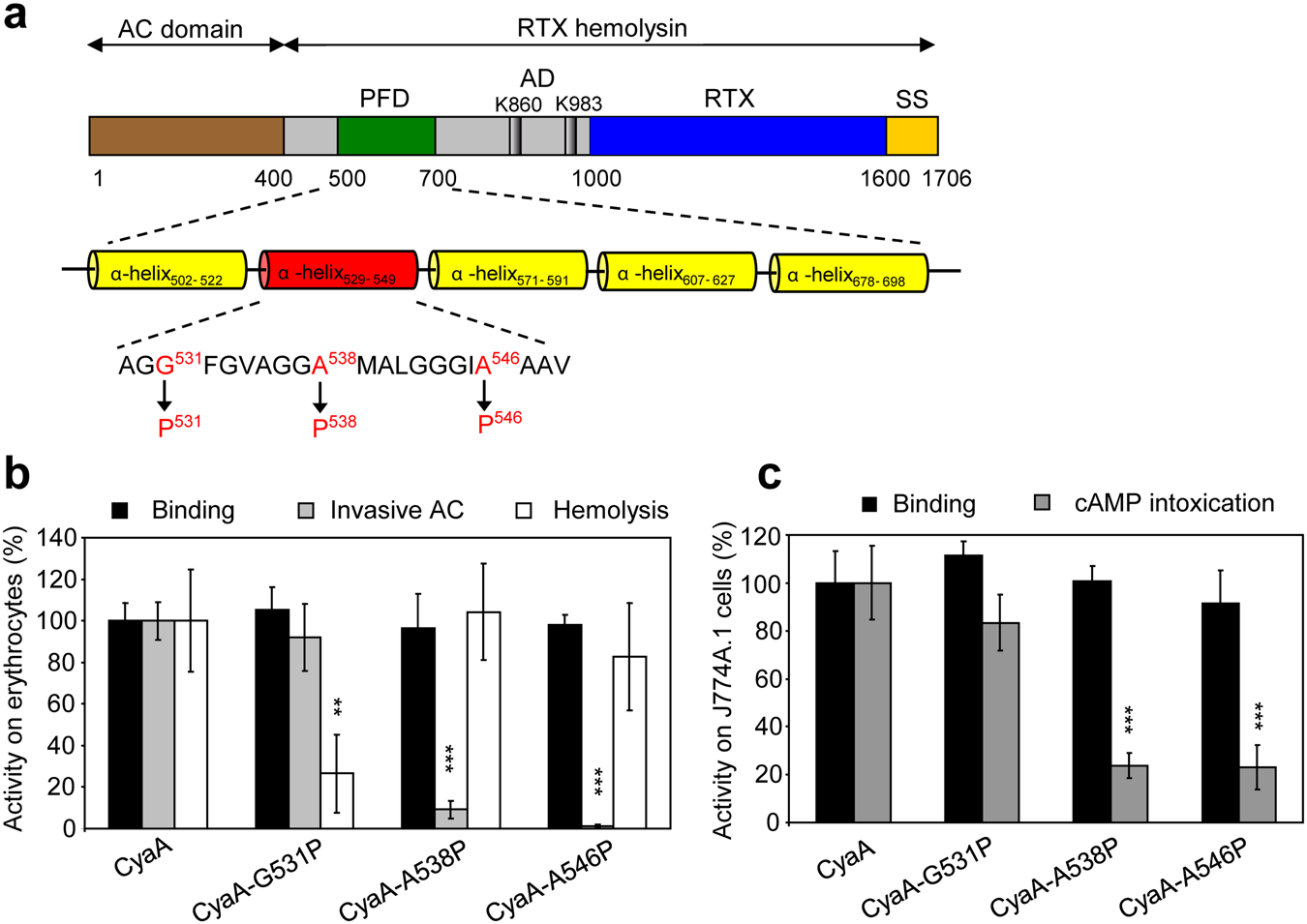
The replacement of glycine or alanine residues in the putative transmembrane α-helix_529–549_ by prolines reduces hemolytic activity (G531P) or AC domain translocation capacity (A538P, A546P) of CyaA. (**a**) Schematic secondary structure of the CyaA molecule with five predicted transmembrane α-helices in the pore-forming domain (residues 500 to 700). Prediction of transmembrane α-helices was performed using the algorithm of Eisenberg *et al*.^37^. Residues of the putative α-helix_529–549_ that were mutated are colored in red. PFD; pore-forming domain, AD; acylated domain, RTX; calcium-binding RTX domain, SS; secretion signal. (**b**) Sheep erythrocytes (RBC, 5 × 10^8^/ml) in 50 mM Tris–HCl (pH 7.4), 150 mM NaCl containing 2 mM CaCl_2_ (TNC buffer) were incubated at 37 °C with 1 μg/ml of CyaA or its mutant variants and after 30 min, aliquots were taken for determinations of the cell-associated AC activity and of the AC activity internalized into erythrocytes and protected against digestion by externally added trypsin. For determination of hemolytic activity, sheep erythrocytes (5 × 10^8^/ml) in TNC buffer were incubated at 37 °C in the presence of 10 μg/ml of CyaA or its mutant variants. Hemolytic activity was measured after 3 h as the amount of released hemoglobin by photometric determination (A_541nm_). (**c**) Binding of wild-type CyaA or its mutant variants to J774A.1 cells (1 × 10^6^) was determined as the amount of cell-associated AC enzyme activity upon incubation of cells with 1 μg/ml of the protein for 30 min at 4 °C. cAMP intoxication was assessed by determining the intracellular concentration of cAMP generated in cells after 30 min of incubation of J774A.1 cells (2 × 10^5^) with four different toxin concentrations from within the linear range of the dose-response curve (100, 50, 25 and 10 ng/ml). (**b**,**c**) Activities are expressed as percentages of wild-type CyaA activity and represent average values ± standard deviations from at least three independent determinations performed in duplicate with at least two different toxin preparations. Significant differences are indicated by asterisks (**p < 0.01; ***p < 0.001).

The adenylate cyclase toxin-hemolysin (CyaA, ACT or AC-Hly) of pathogenic *Bordetellae* is a member of the RTX (Repeats-in-ToXin) protein family and is a key virulence factor of the whooping cough agent. The 1706 residue-long polypeptide (Fig. 1a) comprises an N-terminal adenylate cyclase (AC) domain (~400 residues) that is linked to a pore-forming RTX hemolysin (Hly) moiety of ~1300 residues. The Hly part of the toxin itself comprises (i) an AC-to-Hly linking segment (residues 400–500), involved in AC domain translocation into target cells; (ii) a hydrophobic pore-forming domain (residues ~ 500–700); (iii) a post-translational activation domain (residues ~ 800–1000), where post-translational acylation of lysine residues 860 and 983 occurs; (iv) an RTX calcium-binding domain between residues 1008 and 1590 with characteristic repeats of the consensus sequence X-(L/I/F)-X-G-G-X-G-(N/D)-D and (v) a C-terminal secretion signal^1–7^.

CyaA is a bifunctional toxin, whose two mutually independent enzymatic and pore-forming activities appear to cooperate in maximizing its overall cytotoxic action^8,9^. The dominant activity of the toxin consists in membrane potential-dependent translocation of its AC enzyme domain directly across the cytoplasmic membrane into the cytosol of target cells^10,11^ in a process that is independent of receptor-mediated endocytosis^12^. The AC domain is next activated by binding of cytosolic calmodulin and catalyzes unregulated conversion of ATP to cAMP, thus subverting cellular signaling^13,14^. In parallel, the Hly moiety oligomerizes and forms small cation-selective membrane pores of 0.6–0.8 nm in diameter, which promotes efflux of potassium ions from the cells and can cause colloid-osmotic (oncotic) cell lysis, such as hemolysis of erythrocytes^15–21^. Several independent lines of evidence suggest that these two activities are accomplished by at least two distinct conformers of CyaA, one accounting for AC domain translocation, the other for formation of membrane pores^22–25^.

The primary target cells of CyaA appear to be host myeloid phagocytes, to which the toxin binds with high affinity through the α_M_β_2_ integrin, known as complement receptor 3 (CR3), CD11b/CD18, or Mac-1^26,27^. The initial and rather unspecific interaction with the receptor occurs through N-linked glycans of the CD11b subunit that are recognized by the RTX domain of the toxin. This primary interaction is followed by a highly specific binding of the RTX block II/III segment with the loop containing residues 614 to 682 on the CD11b subunit of the α_M_β_2_ integrin^27–29^. With a reduced efficacy, the CyaA can also bind and penetrate all kinds of non-myeloid cells that do not express the CD11b/CD18 heterodimer, such as erythrocytes or epithelial cells. Due to its extreme AC enzyme activity, the CyaA toxin can then elevate cytosolic cAMP to detectable and potentially physiologically relevant levels also in cells lacking CD11b/CD18^12,30–34^.

The structure of the N-terminal AC enzyme domain and of the C-terminal RTX block V domain of CyaA have been solved by X-ray crystallography^1,35^ and low-resolution SAXS structures of the RTX domain and of CyaA monomers in solution have been obtained^36^. However, the structures of the pore-forming and acylated domains remain elusive. Five putative transmembrane amphipathic α-helices (I_502–522_,II_529–549_,III_571–591,_IV_607–627_ and V_678–698_) could be predicted in the hydrophobic pore-forming domain by the algorithm of Eisenberg^25,37^. We and others previously showed that the predicted helices I, III and IV are essential both for AC domain translocation and for pore-formation by CyaA^22,25,38,39^. The predicted transmembrane helix V then likely takes part only in the pore-forming activity of the toxin^38^. Recently, alanine substitutions of the G530, G533 and G537 residues of a putative glycine cluster in the predicted helix II (α-helix_529–549_) were reported to cause a dramatic reduction of the specific hemolytic activity of a CyaA-Hly construct that lacks the AC domain and most of the AC-to-Hly linking segment of CyaA (deletion of N-terminal 481 residues). The authors concluded that the glycine cluster of the helix II might be a crucial component of the CyaA pore structure^40^.

Therefore, we examined the role of the putative helix II in biological activities of CyaA and show that it is involved in membrane penetration and pore-forming activity of CyaA.

## Results

### Substitutions along the predicted helix II dissociate the AC domain translocating and hemolytic activities of CyaA

To define the role of the predicted transmembrane α-helix_529–549_ (helix II) of CyaA (Fig. 1a) in membrane penetration of the toxin, we constructed CyaA variants with helix-breaking proline substitutions introduced at the beginning (Gly531), in the center (Ala538) and at the end (Ala546) of helix II (Fig. 1a). These CyaA variants were expressed in *E. coli*, purified close to homogeneity and their biological activity was assessed. Sheep erythrocytes were used as model target cells devoid of the CR3 receptor (CD11b^−^ cells) to which CyaA binds on phagocytes. The mouse J774A.1 macrophages were then used as model phagocytic target cells expressing the toxin receptor CR3 (CD11b^+^ cells). As shown in Fig. 1b, the G531P substitution caused a substantial reduction (by ~70%) of the pore-forming (hemolytic) activity of the toxin on sheep erythrocytes, while the binding and AC translocation capacities of the CyaA-G531P toxin to both CD11b^−^ erythrocytes and CD11b^+^ J774A.1 macrophages were indistinguishable from the activities of wild-type CyaA (Fig. 1b,c). In contrast, substitutions of the Ala538 and Ala546 residues by proline residues reduced selectively the capacity of the CyaA-A538P and CyaA-A546P constructs to deliver the AC domain into the cells, while the binding and hemolytic activities of these mutant toxins were comparable to those of CyaA (Fig. 1b,c).

**Figure 2:**
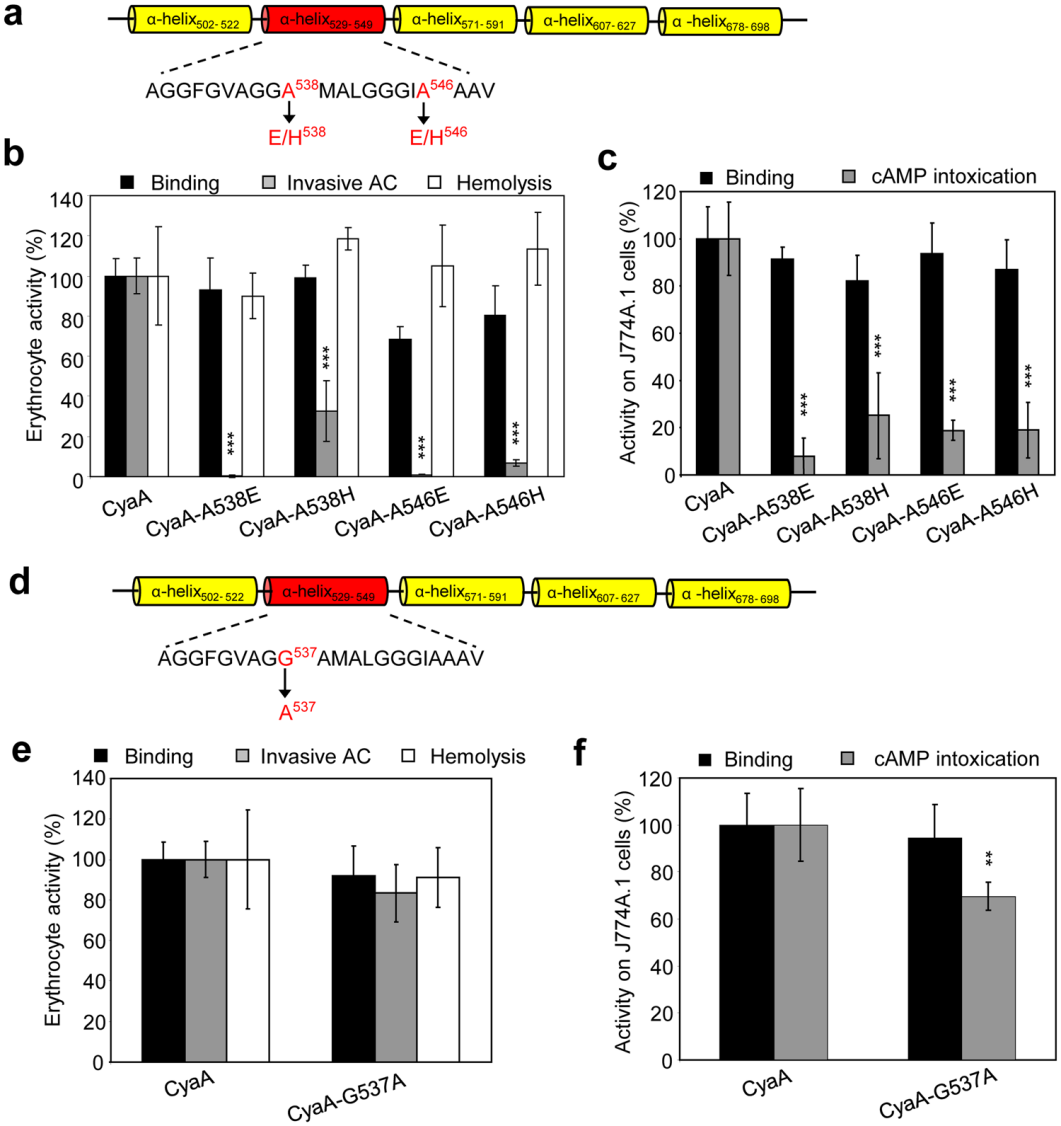
Replacement of the Ala538 and Ala546 residues by histidines and glutamates reduces AC domain translocation but not the hemolytic activity. (**a**) Schematic secondary structure of the predicted transmembrane α-helices in the pore-forming domain of CyaA. Residues of the putative α-helix_529–549_ that were mutated are colored in red. Biological activities of CyaA or its mutant variants were analyzed using CD11b^−^ sheep erythrocytes (**b**) and CD11b^+^ J774A.1 mouse macrophages (**c**). Preparations and analyses of samples were performed as described in the legend to Fig. 1. (**b**,**c**) Activities are expressed as percentages of CyaA activity and represent average values ± standard deviations from at least three independent determinations performed in duplicate with two different toxin preparations. Significant differences are indicated by asterisks (**p < 0.01; ***p < 0.001). (**d**) Schematic secondary structure of the predicted transmembrane α-helices in the pore-forming domain of CyaA. The mutated Gly537 residue in the putative α-helix_529–549_ is colored in red. Biological activities of CyaA or its CyaA-G537A mutant were analyzed using CD11b^−^ sheep erythrocytes (**e**) and CD11b^+^ J774A.1 mouse macrophages (**f**). Preparations and analyses of samples were performed as described in the legend to Fig. 1. Activities are expressed as percentages of CyaA activity and represent average values ± standard deviations from at least three independent determinations performed in duplicate with two different toxin preparations. Significant differences are indicated by asterisks (**p < 0.01).

To test if this phenotype was due to helix-breaking properties of the introduced proline residues, we replaced Ala538 and Ala546 by negatively charged glutamates, or by histidine residues that bear a heterocyclic side chain (Fig. 2a). As shown in Fig. 2b,c, the CyaA-A538E variant bound to CD11b^−^ as well as to CD11b^+^ cells with the same efficacy as the wild-type CyaA and the binding of the CyaA-A546E protein to the CD11b^−^ cells was only slightly reduced (by ~30%). While the hemolytic activity of the CyaA-A538E and CyaA-A546E variants remained unaffected (Fig. 2b), their specific AC translocation capacity was selectively impaired (Fig. 2b,c). Similar results were obtained when the A538H and A546H substitutions were introduced into the CyaA molecule (Fig. 2b,c).

In a recent study, an alanine substitution of the Gly537 residue was reported to cause a severe reduction of the hemolytic activity of a truncated CyaA-Hly construct that lacks the N-terminal AC domain and most of the AC-to-Hly linking segment^40^. However, our above described A538P/H/E substitutions of the adjacent residue Ala538 had no impact on the hemolytic activity in the context of the full-length CyaA molecule (*cf*. Figs 1b and 2b). Therefore, we replaced the Gly537 residue by alanine to generate the full-length CyaA-G537A construct (Fig. 2d). As summarized in Fig. 2e,f, binding of the CyaA-G537A protein to erythrocytes and J774A.1 macrophages, as well as its specific hemolytic activity on erythrocytes remained unaltered. Compared to wild-type CyaA, the AC domain translocation capacity of CyaA-G537A was slightly but significantly reduced only on the J774A.1 macrophage target cells. This shows that caution is needed in interpretation of phenotypes of substitutions that are introduced into the truncated CyaA constructs that lack the AC domain and the AC-to-Hly linker. Taken together, our results suggest that the N-terminal part of the helix II segment between residues 529 to 549 is important for the hemolytic activity of CyaA, while the central and C-terminal portions of the predicted helix II appear to participate in AC domain translocation across the plasma membrane of target cells.

### Fully hemolytic CyaA with a substitution within helix II is insensitive to activation of pore formation by membrane potential

**Figure 3:**
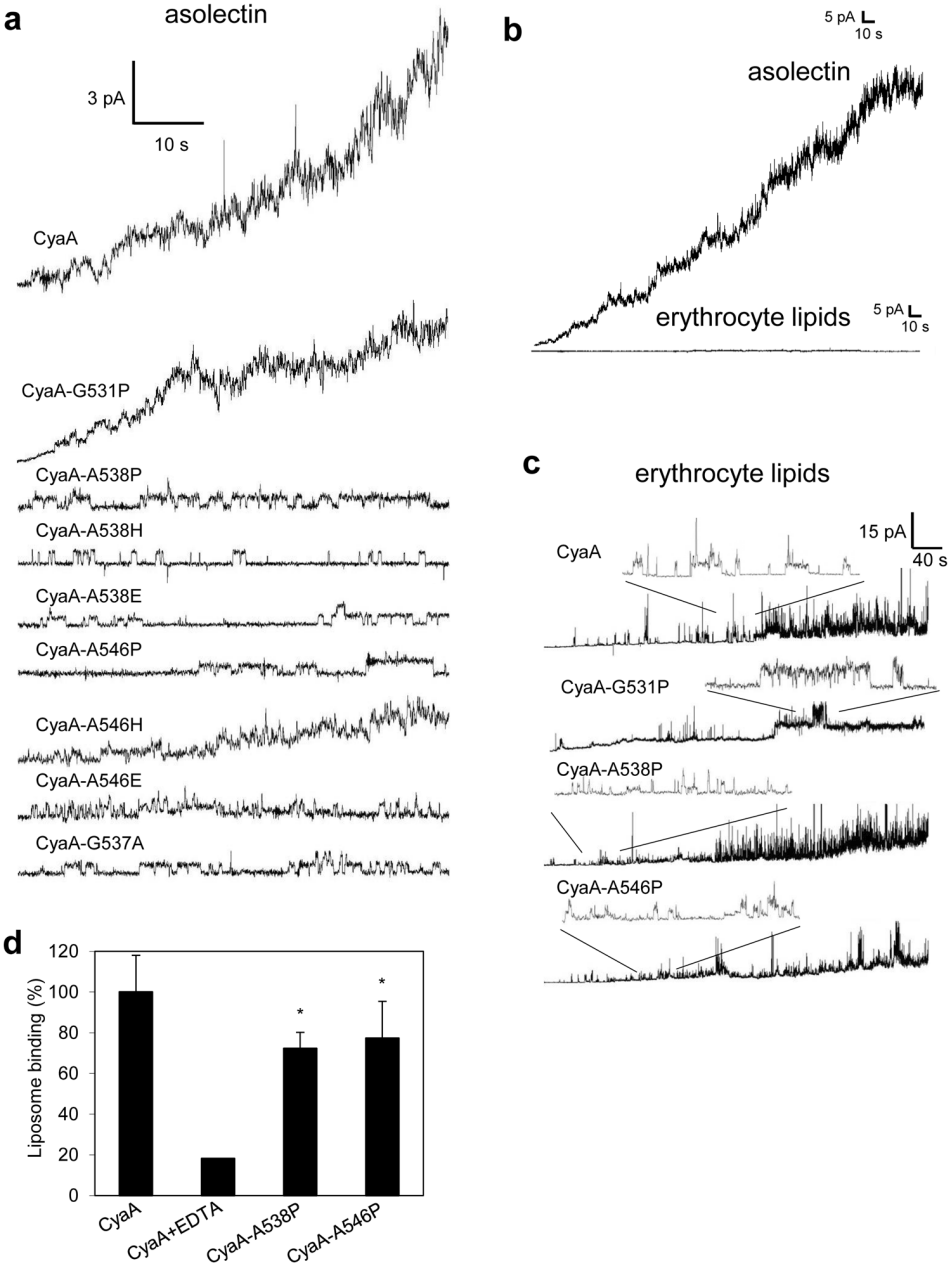
CyaA mutants of Gly537, Ala538 and Ala546 formed importantly lower numbers of open pores over the same time period than CyaA. (**a**) Typical current traces of asolectin membranes exposed to CyaA and its mutants. Measurement conditions on asolectin/decane:butanol (9:1) membranes: 150 mM KCl, 10 mM Tris-HCl (pH 7.4), 2 mM CaCl_2_, CyaA concentration 1 nM, the applied voltage was −50 mV and the temperature was 25 °C. In the figure we show one representative kinetics out of ten performed recordings. Membranes made from sheep erythrocyte lipids are poor targets of CyaA. (**b**) Typical current traces of erythrocyte or asolectin membranes exposed to CyaA. Lipids/decane:butanol (9:1) membranes: 150 mM KCl, 10 mM Tris-HCl (pH 7.4), 2 mM CaCl_2_, CyaA concentration 1 nM, the applied voltage was −50 mV and the temperature was 25 °C. (**c**) Typical current traces of erythrocyte membranes exposed to CyaA and its variants. Measurement conditions on erythrocyte lipids/decane:butanol (9:1) membranes: 1 M KCl, 10 mM Tris-HCl (pH 7.4), 2 mM CaCl_2_, toxin concentration 50 nM, the applied voltage was −83 mV, the temperature was 25 °C. In the figure we show one representative kinetics out of ten performed recordings. Binding capacity of the CyaA-A538P and CyaA-A546P mutants to liposomes made from asolectin is slightly reduced. (**d**) Asolectin 1000 nm liposomes (4 mg/ml) in 50 mM Tris–HCl (pH 7.4), 150 mM NaCl containing 2 mM CaCl_2_ or 5 mM EDTA (negative control) were incubated at 37 °C with 1 μg/ml of CyaA or its mutant variants. After 15 min of incubation, the liposomes were washed, transferred to the fresh tube and lyzed with 0.1% Triton X-100 for determination of membrane-bound AC enzyme activity. Binding capacity of wild-type CyaA in the presence of 2 mM calcium ions was taken as the respective 100% binding activity and represent average values ± standard deviations from two independent determinations performed in duplicate. Significant differences are indicated by asterisks (*p < 0.05).

To corroborate the characterization of CyaA constructs with residue substitutions within the predicted helix II, we first analyzed their overall membrane activity (*e.g*. the product of the number of molecules inserted into membrane and forming pores, their unit conductance and lifetime of open pores). As documented by representative current recordings in Fig. 3a and Supplementary Fig. S1, the CyaA-G531P construct exhibited a reduced overall membrane activity, compared to wild-type CyaA, in agreement with its about three fold reduced hemolytic capacity (*c.f*. Fig. 1b). The CyaA-G531P toxin formed pores with the most frequent single pore conductance of 8.1 pS, which is similar to the conductance of 8.7 pS of pores formed by wild-type CyaA (Table 1 and Supplementary Fig. S2). Previously, we observed that CyaA forms pores exhibiting two different lifetimes within asolectin membranes^6^. The shorter lifetime (τ1 ~ hundreds of ms) likely corresponds to the rapid oscillation between the open and closed state of an already existing pore. The longer lifetime (τ2 > 500 ms) would correspond to formation and disassembly of the oligomeric pores that account for most of the overall membrane conductance produced by CyaA. As documented in Table 1 and Supplementary Fig. S3, the CyaA-G531P mutant formed pores with slightly shorter τ2 (0.73 ± 0.11 s) than CyaA (1.07 ± 0.04 s).

**Table 1:** Activities of CyaA mutants on asolectin planar lipid membranes.

	Most frequent single pore coductance (pS)^a^		Most frequent pore lifetime τ (s)^b^	
	state 1	state 2	τ1	τ2
CyaA	8.7 ± 1.8	N.D.	0.21 ± 0.01	1.07 ± 0.04
CyaA-G531P	8.1 ± 1.9	N.D.	0.22 ± 0.05	0.73 ± 0.11
CyaA-A538P	8.0 ± 2.2	13.0 ± 2.5	0.27 ± 0.01	1.12 ± 0.06
CyaA-A538H	7.2 ± 1.2	8.6 ± 2.1	0.40 ± 0.05	1.27 ± 0.17
CyaA-A538E	6.8 ± 1.3	9.1 ± 2.7	0.21 ± 0.01	1.11 ± 0.07
CyaA-A546P	8.8 ± 2.3	12.4 ± 2.7	0.24 ± 0.01	1.34 ± 0.08
CyaA-A546H	6.1 ± 1.5	N.D.	0.15 ± 0.01	N.D.
CyaA-A546E	6.6 ± 1.4	8.8 ± 1.3	0.26 ± 0.01	N.D.
CyaA-G537A	8.7 ± 1.7	N.D.	0.29 ± 0.04	0.80 ± 0.06

^a^Single-pore conductance of wild-type CyaA and its mutant variants (1 nM) was determined in 150 mM KCl, 10 mM Tris-HCl and 2 mM CaCl_2_ (pH 7.4) at 25 °C and the applied voltage was −50 mV. The average values ± S.D. (half width at half maximum) are shown. The experiments were repeated with at least two independent protein preparations and on more than twenty different membranes.

^b^For lifetime determination, the logarithmic histogram of dwell times (of ~700 individual pore openings) was fitted with a single- or double-exponential function^57^. The error estimates of lifetimes were obtained by bootstrap analysis.

Unexpectedly, the CyaA-A538P/H/E, CyaA-A546P/H/E and CyaA-G537A mutants exhibited a strongly reduced overall membrane activity in asolectin membranes (Fig. 3a and Supplementary Fig. S1), despite their unaffected pore-forming (hemolytic) activity on erythrocytes (*c.f*. Figs 1b, 2b,e). To test if membranes made of biologically more relevant lipid would yield better results than the routinely used soybean asolectin membranes, we compared the pore-forming properties of the toxins on black lipid membranes prepared from lipids extracted from sheep erythrocyte membrane. However, as shown in Fig. 3b, the overall membrane activity of 1 nM CyaA was much lower on bilayers made from erythrocyte lipids, than on membranes made from soybean asolectin (both 3% in decane:butanol). In order to increase the single pore conductance to detectable levels we increased the KCl concentration from 150 mM to 1 M. Moreover, we intended to increase the pore-forming activity by simply elevating the CyaA concentration and increasing the membrane voltage^41^. As shown in Fig. 3c, despite a 50-times higher toxin concentration, increased concentration of KCl (1 M) and a higher applied voltage (−83 mV), still a very low overall membrane activity of CyaA was observed on erythrocyte-derived lipid bilayers (*cf*. Fig. 3a,b). This prevented a direct correlation of the pore-forming activity of the CyaA variants on erythrocytes (hemolytic activity) to that on erythrocyte lipid-derived black lipid membranes.

**Figure 4:**
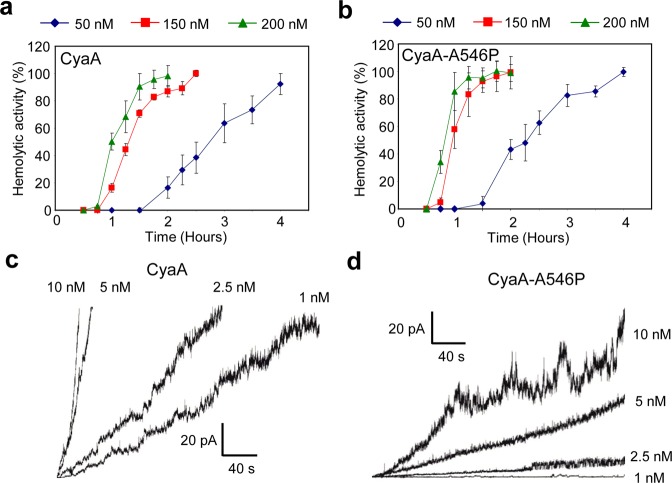
Fully hemolytic CyaA-A546P exhibits approximately 10-times lower specific membrane activity on asolectin bilayers than CyaA. For determination of hemolytic activity, sheep erythrocytes (5 × 10^8^/ml) in TNC buffer were incubated at 37 °C in the presence of 50, 150 and 200 nM of CyaA (**a**) or CyaA-A546P (**b**). Hemolytic activity was measured in time for 4 h as the amount of released hemoglobin by photometric determination (A_541nm_). Activities are expressed as percentages of hemolytic activity and represent average values ± standard deviations from at three independent determinations performed in duplicate. Typical current traces of 1, 2.5, 5 and 10 nM CyaA (**c**) and CyaA-A546P (**d**) on asolectin/decane:butanol (9:1) membranes. Measurement conditions: 150 mM KCl, 10 mM Tris-HCl (pH 7.4), 2 mM CaCl_2_, the applied voltage was −50 mV, the temperature was 25 °C.

The very low membrane activities of the CyaA-A538P and CyaA-A546P proteins with substitutions in helix II were not due to a reduced capacity to bind asolectin membranes. These proteins bound asolectin liposomes almost as well as the wild-type CyaA, exhibiting only ~28 and ~23% lower binding capacity, respectively (Fig. 3d). The discrepancy between the unaffected hemolytic activity of the CyaA-A546P mutant on erythrocytes and its very low overall membrane activity on asolectin membranes was, indeed, observed within a range of protein concentrations. While the CyaA and CyaA-A546P proteins elicited highly comparable times courses of erythrocyte lysis at the three tested protein concentrations of 50, 150 and 200 nM (Fig. 4a,b), there was a striking difference in their membrane activities on asolectin bilayers (Fig. 4c,d). At equal concentration, the wild-type CyaA exhibited approximately 10-times higher membrane activity than CyaA-A546P. However, this was neither due to reduced conductance, nor to decreased lifetime of the individual pores formed by CyaA-A546P (Table 1, Supplementary Figs S2 and S3). In fact, at 1 nM concentration, the CyaA-A538P and CyaA-A546P proteins formed pores with even broader distribution of conductance units (~4–20 pS) than the CyaA (~4–14 pS), or the CyaA-G531P (~4–13 pS) mutant (Table 1 and Supplementary Fig. S2). Moreover, by difference to CyaA, two distinct single pore conducting states were resolved for the CyaA-A538P (8.0 and 13.0 pS) and CyaA-A546P (8.8 and 12.4 pS) constructs (Table 1 and Supplementary Fig. S2). Furthermore, with very low occurrence and in contrast to wild-type CyaA, both CyaA-A538P and CyaA-A546P variants formed also pore units with conductance higher than 50 pS (Supplementary Fig. S4). In addition, pore lifetimes of both mutants were very close to those of CyaA (Table 1 and Supplementary Fig. S3). However, compared to the wild-type CyaA, the CyaA-A538P and CyaA-A546P mutants formed importantly lower numbers of open pores over the same time period (*cf*. Fig. 3a).

**Figure 5:**
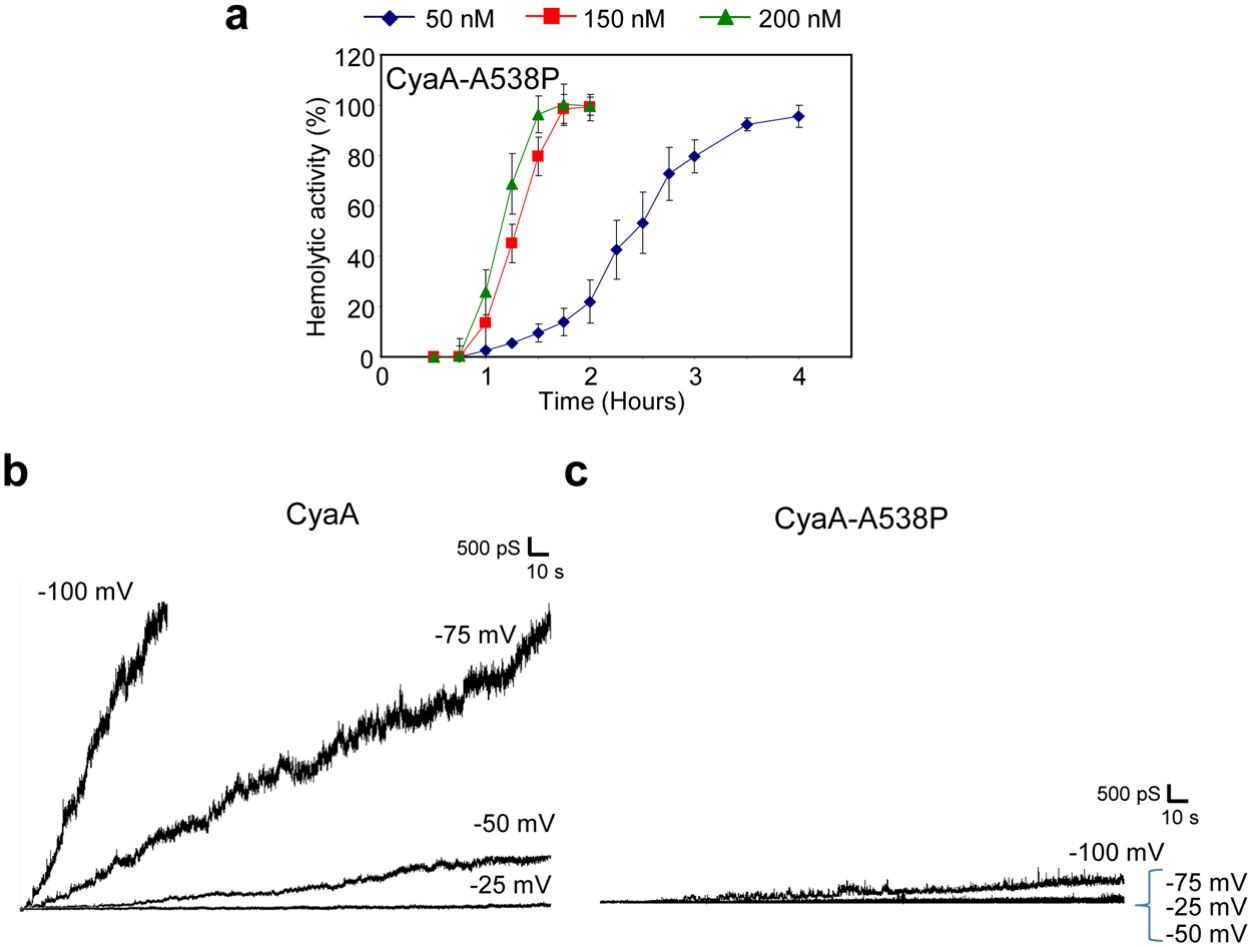
Fully hemolytic CyaA-A538P is insensitive to activation of pore formation by membrane potential. (**a**) For determination of hemolytic activity, sheep erythrocytes (5 × 10^8^/ml) in TNC buffer were incubated at 37 °C in the presence of 50, 150 and 200 nM of CyaA-A538P. Hemolytic activity was measured in time for 4 h as the amount of released hemoglobin by photometric determination (A_541nm_). Activities are expressed as percentages of hemolytic activity and represent average values ± standard deviations from at two independent determinations performed in duplicate. Typical conductance traces of asolectin membranes exposed to CyaA (**b**) or its CyaA-A538P variant (**c**) with different applied membrane potential. Typical conductance trace was calculated from typical current trace (representative of minimum 10 membranes) for each applied membrane potential. Measurement conditions: 150 mM KCl, 10 mM Tris, 2 mM CaCl_2_, pH 7,4; 3% asolectin, 9:1 decane:butanol, toxin concentration 1 nM; applied membrane potential is indicated in the picture.

The observation that wild-type CyaA, CyaA-A546P and CyaA-A538P exhibited equal hemolytic activities at strikingly different overall membrane activities on BLM was perplexing (*c.f*. Figs 1b, 3a, 4a,b and 5a). Among the testable hypotheses for explaining this paradox was the difference in membrane potential in the two systems. The membrane activity assessment on asolectin membranes was systematically performed at −50 mV of applied voltage, while the typical electrical potential on sheep erythrocyte membrane is only around −10 mV. We thus analyzed the membrane activity of CyaA and CyaA-A538P toxins on planar asolectin membranes at different applied voltage. As expected and shown in Fig. 5b, the membrane conductance elicited over time by wild-type CyaA was strongly enhanced in function of the applied voltage, increasing approximately 50-times with increase of the voltage from −25 to −75 mV. The membrane potential of −50 mV was required for efficient formation of CyaA pores and a steady increase of membrane conductance over time. In contrast, there was no increase in conductance observed for the CyaA-A538P mutant at voltage increasing from −25 to −50 or −75 mV. A voltage of −100 mV was needed to enforce a well observable increase of membrane conductance over time for the CyaA-A538P protein (Fig. 5c). This might explain why no difference between hemolytic activities of the wild-type CyaA and CyaA-A538P toxins was seen on erythrocytes that typically bear a rather low membrane potential of about −10 mV^42^, which would not enhance the pore-forming activity of wild-type CyaA any importantly over that of CyaA-A538P.

## Discussion

**Figure 6:**
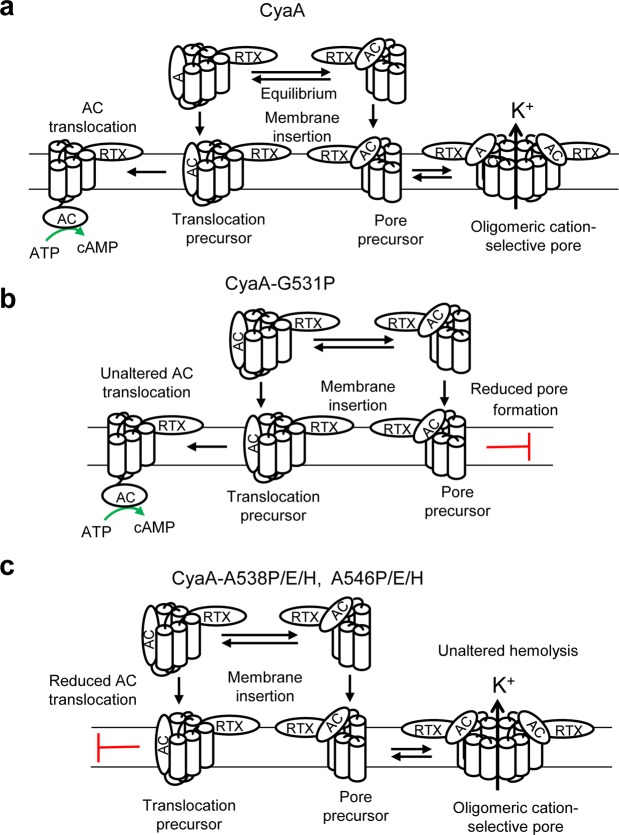
Schematic model of CyaA action on target membrane. (**a**) CyaA penetrates the cytoplasmic membrane and would employ two distinct conformers to accomplish its multiple actions within the membrane. One conformer would form cation-selective pores that permeabilize the membrane bilayer for efflux of cytosolic potassium ions. The other conformer would translocate the AC enzyme domain into cells across the plasma membrane and catalyze conversion of cytosolic ATP to cAMP. (**b**) The CyaA-G531P mutant fails to form membrane permeabilizing pores, but it translocates the AC enzyme domain into the cell cytosol in the same manner as the wild-type toxin. (**c**) The CyaA variants with the substitution of Ala538 and Ala546 fail to translocate the AC domain into the cytosol, but are still capable of forming the membrane permeabilizing pores in the erythrocyte membrane.

In this study, we have focused on the role of the predicted helix II of the pore-forming domain of CyaA and characterized its involvement in penetration of CyaA across the plasma membrane of target cells. We report that a helix-breaking proline substitution of the Gly531 residue at the N-terminal end of the putative helix II selectively impairs the hemolytic activity of CyaA-G531P, without affecting any importantly its specific AC translocation activity. In contrast, proline substitutions of the Ala538 and Ala546 residues in the middle, or at the C-terminal end of the predicted helix II, respectively, selectively impaired the capacity of CyaA to translocate its AC domain across the membrane of target cells, not impacting any importantly on its hemolytic activity. This dissociation of the two membrane activities of the toxin by single residue substitutions within helix II lends further support to the previously proposed model of CyaA action on the membrane of target cells (Fig. 6). This model is based on similar dissociating effects of certain residue substitutions in helices I, III and IV, that selectively affected only one of the two CyaA toxin activities. It postulates that AC enzyme translocation and CyaA pore formation are two distinct activities of CyaA within the target cell membrane and are accomplished in parallel by two distinct types of CyaA conformers^22–25^.

The CyaA-A538P and CyaA-A546P mutants exhibited a strongly reduced AC domain translocation capacity and formed pores with two distinct most frequent single pore conducting states. The substantial decrease, or complete elimination of AC domain translocation capacity of CyaA was also observed when the Ala538 and Ala546 residues were replaced with glutamate and histidine residues, which are not α-helix breaking residues. This indicates that the Ala538 and Ala546 are part of a key structural segment involved in AC domain translocation (Fig. 6). Similar reduction of the AC domain translocating capacity has been previously observed upon introduction of a helix-breaking proline residue within three of the four previously characterized transmembrane α-helices of CyaA^22,25,38^. This would indicate that these predicted helices, namely helix I_502–522_, II_529–549_, III_571–591_ and IV_607–627_, are all cooperating in the course of translocation of the invasive AC domain across the plasma membrane of target cells. Moreover, introduction of glutamate or histidine residue substitutions at the end of the predicted helix II (A546E/H mutants) yielded a previously not observed loss of the longer living pore states. In contrast, the proline substitution of the Gly531 residue at the N-terminal end of helix II did not affect the AC domain translocation capacity of the CyaA-G531P construct any importantly but reduced significantly the specific hemolytic activity of the CyaA-G531P mutant. Hence, the Gly531 residue appears to be important for the pore-forming activity of CyaA, while the Ala538 and Ala546 residues are crucial for the AC domain translocation capacity. These differential effects of substitutions of the Gly531, Ala538 and Ala546 residues may, however, also indicate that these residues are not part of the same α-helical structure. This was, indeed, predicted earlier by Powthongchin *et al*.^39^ who used the PHDhtm algorithm that combines a neural network algorithm with multiple alignments^43^. On the contrary, the Eisenberg algorithm^37^ that predicts transmembrane helices on the basis of their calculated average hydrophobicity, predicted the Gly531, Ala538 and Ala546 residues to be part of the same transmembrane α-helix^25^. Currently a number of algorithms for prediction of transmembrane helices is available^44^. However, in the absence of any structural information on the pore-forming domain of CyaA, any reliable prediction of its overall organization and the delimitation and topology of its individual transmembrane helices remains difficult.

Artificial lipid bilayers made of asolectin differ from mammalian cell membranes in lipid composition, presence of solvent and thickness. Nevertheless, asolectin bilayers have been successfully used for decades for measurements of pore characteristics of various proteins, including CyaA mutant variants^6,16,22,25,38,45^. In these black lipid membrane experiments the CyaA was shown to interact with target membranes and form pores within seconds^15,16,46^. As also demonstrated earlier, the polarity of the voltage with respect to the addition of CyaA determines the type of pores formed and their voltage dependence^41^. Moreover, data from osmotic protection experiments suggest that within erythrocyte membrane the CyaA forms a pore of 0.6 and 0.8 nm in diameter^17^. The CyaA pore likely arises by insertion and subsequent aggregation of the CyaA monomers into higher order oligomers^20^. Opening of these pores permeabilizes cells for potassium efflux and can eventually lead to colloid-osmotic (oncotic) lysis due to equilibration of ions through the pore, resulting in a net inflow of ions, influx of water, cell swelling and erythrocyte lysis within hours.

We report that for CyaA variants with substitutions of the Gly537, Ala538 and Ala546 residues the overall pore-forming activity on asolectin membranes was very low. At the same time these proteins exhibited a normal hemolytic activity on erythrocytes. A detailed characterization of single pores formed by these CyaA constructs demonstrated that the observed decrease of overall membrane activity of these proteins on asolectin membranes was mainly due to their substantially reduced sensitivity to activation of pore formation by the applied voltage (membrane potential). The moderate changes in single-pore conductances and lifetimes of the rare pores formed by these toxin variants cannot explain their strikingly decreased overall activity on asolectin membranes. A small effect could, perhaps, be also attributed to a slightly reduced asolectin membrane binding capacity, which was observed for the CyaA-A538P and CyaA-A546P variants on liposomes made of asolectin. Moreover, with low occurrence, the CyaA-A538P and CyaA-A546P variants formed in asolectin bilayers even larger pores than the wild-type CyaA. The occurrence of larger pores, although at a very low frequency, may contribute to comparable kinetics of oncotic lysis of erythrocytes by the mutant and wild-type CyaA. The formation of bigger pores combined with higher frequency of pore formation leading to “hyperhemolytic” phenotype was then recently described after substitutions of negatively charged residues located in the AC-to-Hly linker segment of CyaA^6^. The AC-to-Hly segment together with the structure containing Gly537, Ala538 and Ala546 may, hence, interact together and control the formation of CyaA pores with restricted pore size.

We prepared also artificial membranes from lipids isolated from sheep erythrocytes. However, the overall activity of CyaA on this type of membranes was very low and did not allow analysis of the differences between the activities of CyaA mutant. It remains unclear why the overall membrane activity of CyaA on planar membranes made from erythrocyte lipids was that low in comparison to CyaA activities on membranes made from asolectin. A plausible hypothesis would be that asolectin, a mixture of lipids extracted from soybean, may contain minor lipid(s) that destabilize the lipid packing of the bilayer and thereby facilitate the insertion of the toxin molecule into the membrane. Indeed, the comparison of lipid composition of type II asolectin from soybeans with the composition of lipids extracted from sheep erythrocyte membrane revealed that the contents of individual lipids differ significantly. In line with the previous study^47^, soybean asolectin was mainly composed of neutral phosphatidylcholine and phosphatidylethanolamine (45.7% and 22.3%, respectively) and it contained ~31% of negatively charged lipids (phosphatidylserine, phosphatidylinositol, phosphatidic acid, Supplementary Table 1), possibly containing minor amounts of phytosterols^48^. By difference, sheep erythrocyte lipids contain a high amount of cholesterol (~30% of total lipids)^49^. Phosphatidylethanolamine then accounted for 64% of the total erythrocyte phospholipids and phosphatidylserine, phosphatidylcholine and sphingomyelin were present in lesser amounts (Supplementary Table 2). It is known that lipid composition has a very strong effect on stability of transmembrane helices and protein oligomerization within the membrane^50^. The lipid composition of target membranes was also shown to play an important role in CyaA activity. For example phosphatidylethanolamine, a lipid known for inducing negative membrane curvature and formation of non-bilayer (hexagonal) phases^51^, enhanced membrane activity of CyaA^32^. Even though CyaA does not contain functional cholesterol binding motifs^38^, the cholesterol content of the membrane was found to modulate penetration of CyaA across cellular membrane^32,52,53^, likely by lowering of the energy barrier controlling insertion of the transmembrane helices and subsequent AC domain translocation across the lipid bilayer.

It is plausible to speculate that the helix II structure, comprising residues Gly537, Ala538 and Ala546, may play an important role in the association/dissociation equilibrium involved in CyaA oligomerization^20,40^. Our data further goes well with the report of Prangkio *et al*., showing that a synthetic peptide corresponding to the putative α-helix II of CyaA (Trp528-Gly550) can permeabilize the membrane of liposomes made from phosphatidylcholine, phosphatidylethanolamine and cholesterol and of sheep erythrocytes^54^. While the conserved Tyr940 residue from the acylated domain of CyaA appears to initiate the first steps of membrane insertion of CyaA^38^, the structure containing the Gly537, Ala538 and Ala546 residues might next be involved in the ability of CyaA to incorporate into membranes of target cells, contributing to protein-lipid interaction.

Based on the herein reported data we propose a schematic model (Fig. 6), where the residues 529 to 549 of helix II are predicted to participate in AC domain translocation and pore formation by CyaA. While the G531P substitution would selectively interfere with the oligomerization of CyaA pore precursors and thereby reduce hemolytic activity without affecting AC domain translocation, the single substitutions of the A538 and A546 residues would selectively disrupt structure involved in AC domain translocation by CyaA.

## Methods

### Construction, production and purification of CyaA proteins

The pCACT3 plasmid was used for co-expression of *cyaC* and *cyaA* genes allowing production of recombinant CyaC-activated CyaA in *Escherichia coli*^55^. Oligonucleotide-directed PCR mutagenesis was used to construct pCACT3-derived plasmids for expression of CyaA mutant variants. The cells were grown at 37 °C in MDO medium (yeast extract, 20 g/l; glycerol, 20 g/l; KH_2_PO_4_, 1 g/l; K_2_HPO_4_, 3 g/l; NH_4_Cl, 2 g/l; Na_2_SO_4_, 0.5 g/l; thiamine hydrochloride, 0.01 g/l) supplemented with 150 μg/ml of ampicillin, induced at OD_600_ = 0.6 with 1 mM isopropyl 1-thio-β-D-galactopyranoside (IPTG), and grown for additional 4 h. Then the cells were collected, disrupted by ultrasound, and the insoluble cell debris was extracted with TU buffer (50 mM Tris-HCl (pH 8.0), 8 M urea) containing 0.2 mM CaCl_2_. The CyaA proteins were purified by ion-exchange chromatography on DEAE-Sepharose followed by hydrophobic chromatography on Phenyl-Sepharose as previously described^6^.

### Cell binding, cell invasive and hemolytic activities on sheep erythrocytes

AC enzymatic activities were measured in the presence of 1 µM calmodulin as previously described^56^. One unit of AC activity corresponds to 1 µmol of cAMP formed per min at 30 °C, pH 8.0. Hemolytic activity was measured by determining the hemoglobin release in time upon toxin incubation with 5 × 10^8^/ml washed sheep erythrocytes (LabMediaServis, Czech Republic) as previously described^30^. Cell invasive AC was measured as previously described^6^, by determining the AC protected against externally added trypsin upon internalization into sheep erythrocytes. Erythrocyte binding of the toxins was determined as previously described^6^, by determining the amount of cell-associated AC activity (membrane-bound CyaA). Activity of CyaA was taken as 100%.

### Binding and cAMP elevation of CyaA on J774A.1 cells

Murine monocytes/macrophages J774A.1 (ATCC, number TIB-67) were cultured at 37 °C in a humidified air/CO_2_ (19:1) atmosphere in RPMI medium supplemented with 10% (v/v) heat-inactivated fetal bovine serum (FCS), penicillin (100 I.U./ml), streptomycin (100 µg/ml) and amphotericin B (250 ng/ml). Prior to assays, RPMI was replaced with D-MEM medium (1.9 mM Ca^2+^) without FCS and the cells were allowed to rest in D-MEM for 1 h at 37 °C in a humidified air/CO_2_ (19:1)^8^. J774A.1 cells (10^6^) were incubated in D-MEM with 1 μg/ml of CyaA variants for 30 min at 4 °C, prior to removal of unbound toxin by three washes in D-MEM. After the transfer to the fresh tube, the cells were lyzed with 0.1% Triton X-100 for determination of cell-bound AC enzyme activity. For intracellular cAMP assays, 2 × 10^5^ cells were incubated at 37 °C with CyaA for 30 min in D-MEM, the reaction was stopped by addition of 0.2% Tween-20 in 100 mM HCl, samples were boiled for 15 min at 100 °C, neutralized by addition of 150 mM unbuffered imidazole and cAMP was measured by a competitive immunoassay as previously described^6^. Activity of CyaA was taken as 100%.

### CyaA binding to liposomes

Large unilamellar vesicles (LUVs) of mean size of 1000 nm were prepared by extrusion of multilamellar hand-shaken liposome vesicles made from asolectin (L-α lecithin, Type II S, Sigma) in TN buffer (50 mM Tris–HCl (pH 7.4), 150 mM NaCl) containing 2 mM CaCl_2_, using the LiposoFast Basic apparatus (Avestin) with a polycarbonate membrane of 1000-nm pore diameter (Avestin). The CyaA proteins were diluted from 100 times concentrated stocks to a final concentration of 1 μg/ml within 1 ml of liposome suspensions (LUV 1000 nm) containing 4 mg of lipid in TN buffer and containing either 2 mM CaCl_2_ or 2 mM EDTA (control). After 15 min of incubation at 37 °C, the liposomes were washed twice in TN buffer containing 5 mM EDTA and once in 0.1 M Na_2_CO_3_ (pH 10.5). After the transfer to the fresh tube, the liposomes were lyzed with 0.1% Triton X-100 for determination of liposome-bound AC enzyme activity^33^. Binding capacity of CyaA in the presence of 2 mM calcium ions was taken as the respective 100% binding activity values.

### Lipid extraction from red blood cells

Erythrocytes (RBC) were isolated from sheep blood (LabMediaServis, Czech Republic) by centrifugation and washed in 0.9% saline (w/v). Phospholipids were extracted with chloroform-2-propanol mixture (7:11, v/v) after hemolysis using distilled water. After evaporation of the solvent in vacuum at 40 °C, the phospholipids were dissolved in chloroform, filtered through glass fiber filters (Whatman) and concentrated under a stream of nitrogen. The lipid samples were stored at −80 °C and used for measurements on planar lipid bilayer.

### Lipid bilayer experiments

Measurements on planar lipid bilayers (black lipid membranes) were performed in Teflon cells separated by a diaphragm with a circular hole (diameter 0.5 mm) bearing the membrane. The CyaA toxin was diluted in TU buffer and added into the grounded cis compartment while the trans compartment had a negative electrical potential. This setup corresponds to the situation *in vivo* where the toxin is added outside the cells. For each protein at least two independent preparations were tested on >20 different membranes. The membrane was formed by the painting method using soybean lecithin or erythrocyte lipids in n-decane–butanol (9:1, v/v). Both compartments contained 150 mM or 1 M KCl, 10 mM Tris-HCl (pH 7.4), and 2 mM CaCl_2_, the temperature was 25 °C. The membrane current was registered by Ag/AgCl electrodes, amplified by an LCA-200–100G amplifier (Femto), and digitized by use of a KPCI-3108 card (Keithley) with 1 kHz sampling rate. For determination of most frequent single pore conductances, the conductance histograms (Supplementary Fig. 2) were fitted with one or two Gaussian functions. The function center and half-width at half-maximum are shown in Table 1. To determine pore lifetimes, approximately 700 of individual pore openings were recorded and the dwell times were determined using QuB software with 10 Hz low-pass filter (Gaussian filter in the time domain). The logarithmic histogram of dwell times was constructed with 40 bins per decade and fitted with single- or double-exponential function^57^ using Gnuplot software. The relevant model was selected by the χ2 value. The error estimates of lifetimes were obtained by the bootstrap analysis.

### Thin layer chromatography

Soy and sheep erythrocyte phospholipids (1 mg in chloroform) were separated by thin-layer chromatography (TLC, silica gel 60 G plates; Merck) in chloroform-methanol-water (65:25:4, v/v/v) mobile phase. The spots were detected with iodine vapor and the plates were treated with a 0.2% ninhydrin solution (butanol-acetic acid 95:5, v/v) to localize the amino groups. The spots were collected from the plates, digested with perchloric acid (180 °C, 20 min) and phospholipid amounts were quantified as inorganic phosphate^58^.

### Statistical analysis

Statistical analysis was performed by one-way ANOVA followed by Dunnett’s post-test, comparing all the samples with the control. Significant differences are indicated by asterisks (*p < 0.05; **p < 0.01; ***p < 0.001).

## Supplementary information

Supplementary info

## Data Availability

All data generated or analysed during this study are included in this published article (and its Supplementary Information files).
