# Supplementary material for: Residues 529 to 549 participate in membrane penetration and pore-forming activity of the Bordetella adenylate cyclase toxin
Source: Sci Rep. 2019 Apr 8;9:5758. doi: 10.1038/s41598-019-42200-2 (PMC6453906; doi:10.1038/s41598-019-42200-2)
Supplement: Supplementary file 1 — Supplementary info [file 41598_2019_42200_MOESM1_ESM.pdf]

Supplementary Information

Residues 529 to 549 participate in membrane penetration and pore-forming activity of the *Bordetella* adenylate cyclase toxin

Jana Roderova, Adriana Osickova, Anna Sukova, Gabriela Mikusova, Radovan Fiser, Peter Sebo, Radim Osicka and Jiri Masin

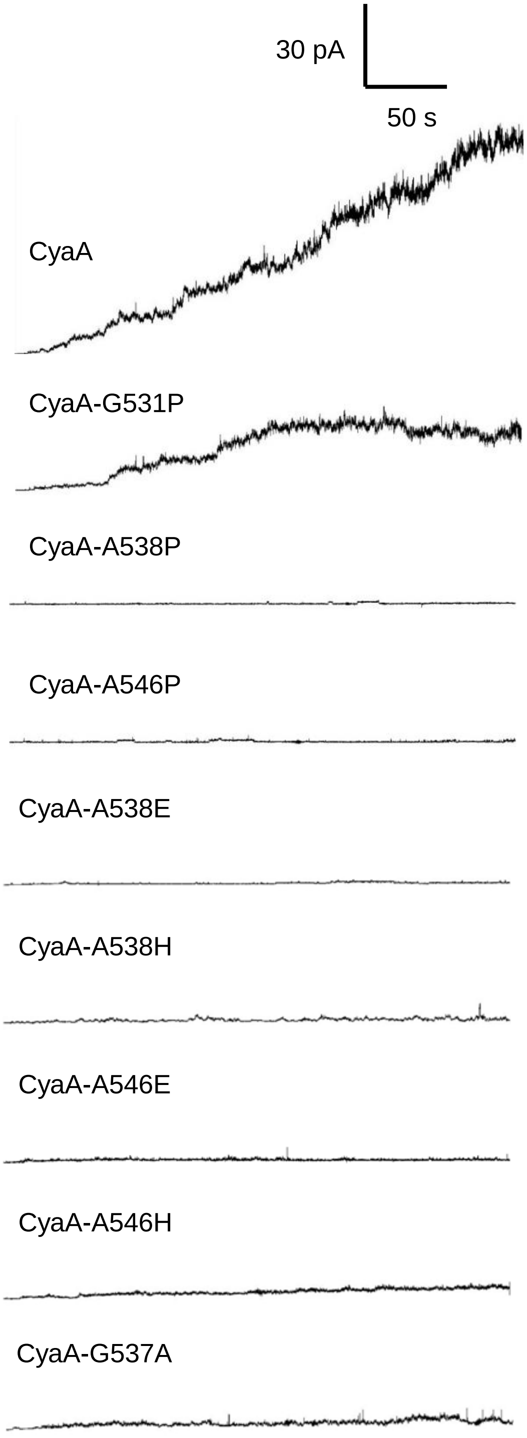

Supplementary Figure S1. Overall membrane activities of wild-type CyaA and its mutant variants on asolectin/decane:butanol (9:1) membranes. Measurement conditions: 150 mM KCl, 10 mM Tris-HCl (pH 7.4), 2 mM $CaCl_2$, toxin concentration 1 nM. The applied voltage was -50 mV and the temperature was 25°C. In the figure we show one representative kinetics out of ten performed recordings.

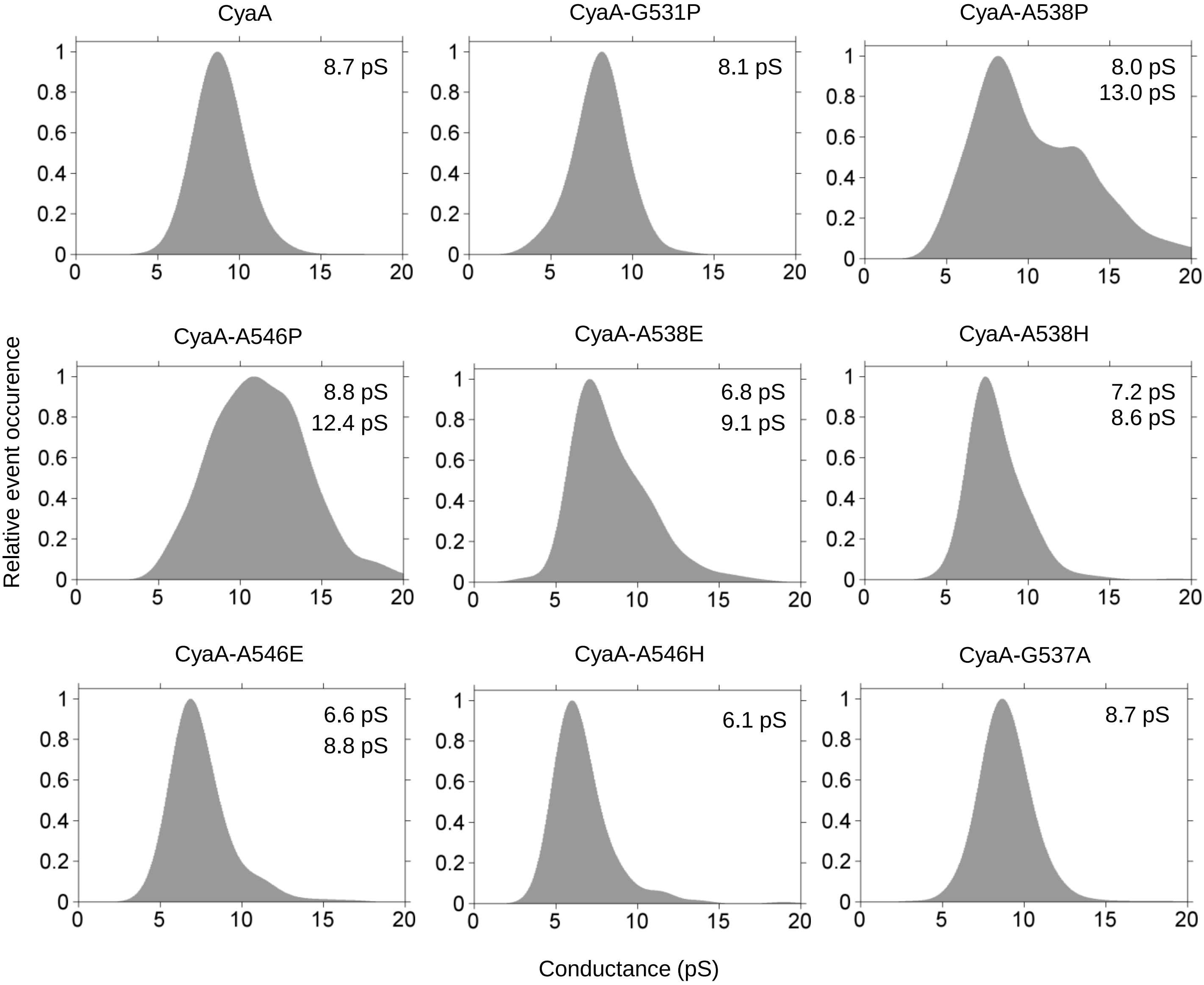

Supplementary Figure S2. Kernel density estimation of single pore conductance of CyaA and mutants in putative transmembrane α-helix$_{529\text{-}549}$ measured on asolectin membranes. Single-pore conductance of wild-type CyaA and its mutant variants (1 nM) was determined in 150 mM KCl, 10 mM Tris-HCl and 2 mM $CaCl_2$ (pH 7.4) at 25°C and applied voltage was -50 mV.

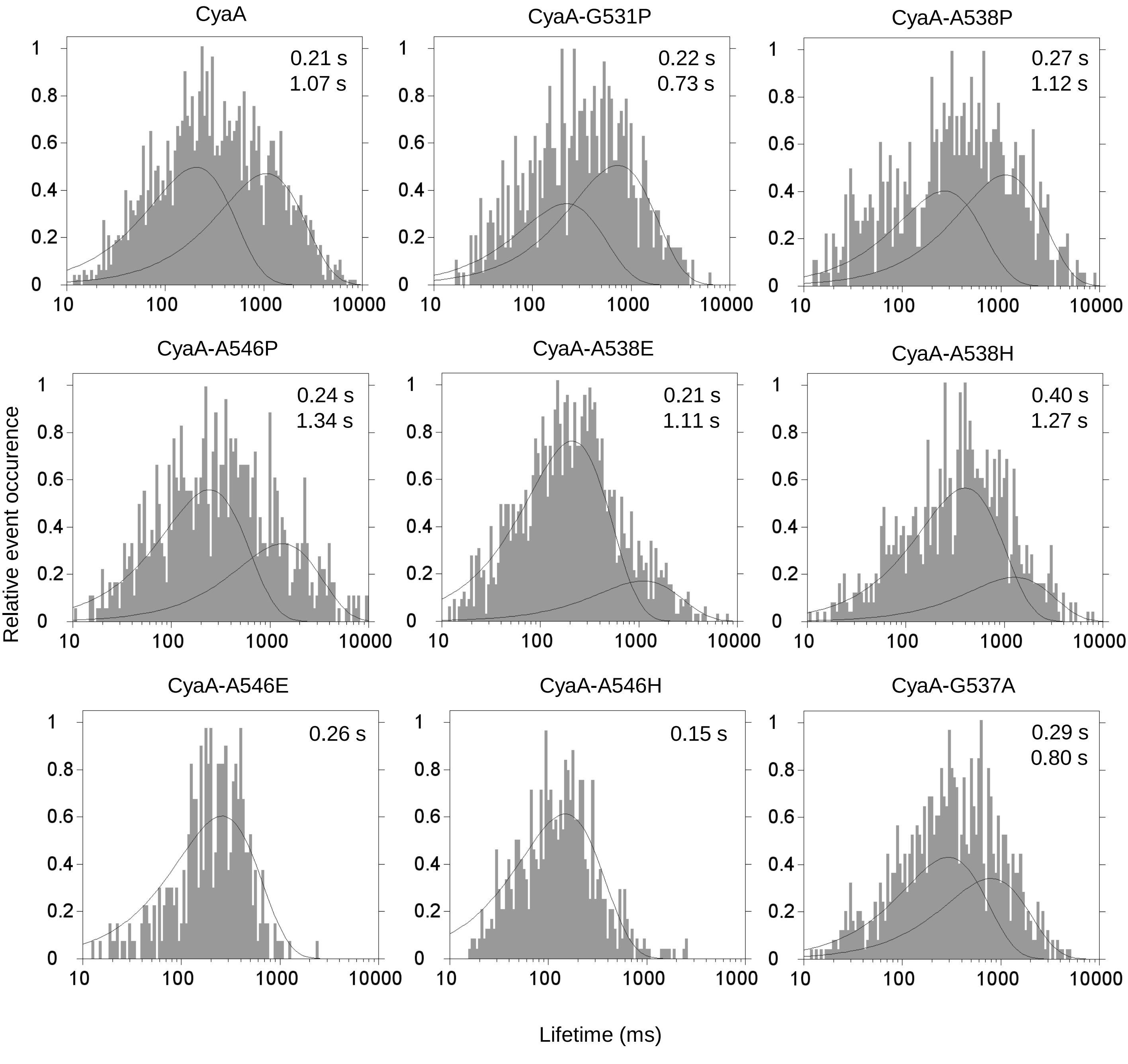

Supplementary Figure S3. Logaritmic histograms of lifetimes of wild-type CyaA and its mutants in putative transmembrane α-helix$_{529\text{-}549}$ measured on asolectin membranes. For lifetime determination, the logarithmic histogram of dwell times (of ~700 individual pore openings) was fitted with a single- or double-exponential function. Measurement conditions: 150 mM KCl, 10 mM Tris-HCl (pH 7.4), 2 mM $CaCl_2$, the applied voltage was -50 mV and the temperature was 25°C.

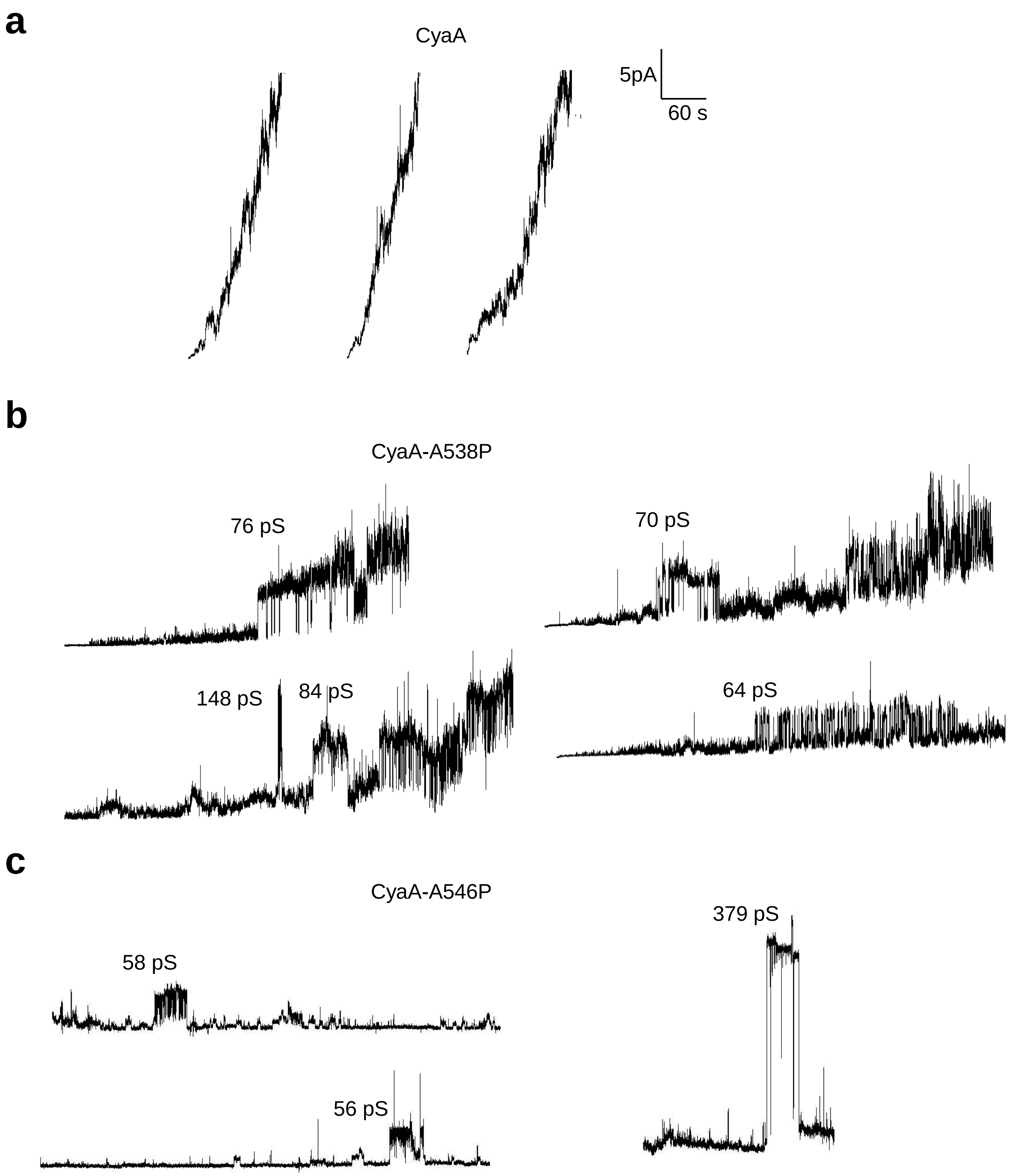

Supplementary Figure S4. With very low occurrence, CyaA-A538P and CyaA-A546P variants formed much bigger pores than wild-type CyaA. Current traces of asolectin membranes exposed to CyaA (**a**) and its CyaA-A538P (**b**) and CyaA-A546P (**c**) mutants. Measurement conditions on asolectin/decane:butanol (9:1) membranes: 150 mM KCl, 10 mM Tris-HCl (pH 7.4), 2 mM $CaCl_2$, CyaA concentration 1 nM, the applied voltage was -50 mV and the temperature was 25°C.

Supplementary Table 1. Table of lipid content of asolectin (type IIS).

	Asolectin phospholipid content (%)[a]
Phosphatidylserine	26.4 ± 2.6
Phosphatidylinositol	
Phosphatidylethanolamine	22.3 ± 0.3
Phosphatidylcholine	45.7 ± 0.4
Phosphatidic acid	4.7 ± 1.8

[a]Soybean phospholipids were separated by thin-layer chromatography in chloroform-methanol-water and finally quantified by means of inorganic phosphate as described in detail in Methods.

Supplementary Table 2. Table of lipid content of sheep erythrocytes.

	RBC phospholipid content (%)
Phosphatidylserine	9.7 ± 2.6
Sphingomyelin<br>Phosphatidylcholine	26.2 ± 1.7
Phosphatidylethanolamine	64.1 ± 4.4

[a]Sheep erythrocyte phospholipids were separated by thin-layer chromatography in chloroform-methanol-water and finally quantified by means of inorganic phosphate as described in detail in Methods.
